# 

**Published:** 1879-01

**Authors:** 


CONTENTS.

| | Page | | Page |
|---|---|---|---|
| Health Maxims | 687 | Health of Soldiers and Clergymen | 699 |
| Health is a Duty | 687 | Tobacco—Its Uses and Abuses | 699 |
| Clarifying Water | 694 | Drowning | 701 |
| A Shake Up | 694 | Consumption | 702 |
| A Happy Home | 695 | Miasm | 704 |
| Lead Colic | 697 | Cellars in Dwellings | 711 |
| The Teeth | 698 | Constipation | 715 |

